# Microstructural injury to the optic nerve with vigabatrin treatment in West syndrome: A DTI study

**DOI:** 10.1038/s41598-025-06336-8

**Published:** 2025-07-16

**Authors:** Junjie Hu, Li Chen, Gongwei Zhang, Yu Fang, Huiting Zhang, Yilian Li, Jianxiang Liao, Cailei Zhao

**Affiliations:** 1 Department of Pediatric, Sihui People’s Hospital, Zhaoqing, China; 2 Department of Neurology, Shenzhen Children’s Hospital, 7019 Yitian Road, Lianhua Street, Futian, Shenzhen, 518000 China; 3 Department of Imaging, Shenzhen Children’s Hospital, 7019 Yitian Road, Lianhua Street, Futian, Shenzhen, 518000 China; 4 Shenzhen Children’s Hospital, China Medical University, Shenzhen, China; 5 Department of Epilepsy Surgery, Shenzhen Children’s Hospital, Shenzhen, China; 6 Shenzhen Children’s Hospital, Medical College of Shantou University, Shenzhen, China

**Keywords:** Vigabatrin, Nuclear magnetic resonance, Cytotoxic oedema, Optic nerve integrity, Neuroscience, Neurology

## Abstract

To evaluate optic nerve injury associated with vigabatrin treatment in children with West syndrome using diffusion tensor imaging. Thirty-five children with West syndrome (aged 9 days–22 months) were retrospectively analyzed and grouped as follows: (1) vigabatrin with symmetrical thalamic abnormalities, (2) vigabatrin without thalamic abnormalities, and (3) controls on other anti-seizure medications. Fractional anisotropy and apparent diffusion coefficient values of the optic nerves were assessed. ROC curves were used to determine fractional anisotropy thresholds for optic nerve injury. fractional anisotropy values in group 1 were significantly lower than those in the control group (P < 0.05), while apparent diffusion coefficient values showed no significant differences. fractional anisotropy values increased significantly after vigabatrin discontinuation (P < 0.05). ROC analysis yielded an fractional anisotropy cut-off value of 304 with 63.6% sensitivity and 100% specificity. fractional anisotropy values are a sensitive imaging biomarker for detecting vigabatrin-related optic nerve injury in West syndrome, particularly when thalamic abnormalities are present. These changes appear reversible after stopping vigabatrin.

## Introduction

Vigabatrin (VGB) is a widely used anti-seizure medication (ASM) for the treatment of West syndrome. It functions as an irreversible inhibitor of γ-aminobutyric acid (GABA) transaminase^1^, thereby preventing the breakdown of GABA and increasing its concentration in the brain^2–4^. This mechanism underlies its proven efficacy in controlling epileptic spasms^5,6^. However, VGB has been associated with visual adverse effects, affecting approximately one-third of treated children^7^. These effects—commonly referred to as VGB-induced retinopathy—are characterized by retinal toxicity that can lead to localized functional deficits and structural atrophy. In adult patients, studies using electroretinography (ERG) and optical coherence tomography (OCT) have revealed thinning of the retinal nerve fiber layer and various forms of retinal cell alterations^8,9^

VGB has demonstrated a seizure-free efficacy rate of approximately 61.8% and is currently recommended as a first-line treatment for West syndrome^5^. However, evaluating VGB-related visual impairment in children remains a clinical challenge. In older children (typically over 9 years) with sufficient cognitive ability, visual field testing such as perimetry can be performed. Flash visual evoked potentials (FVEP) are applicable in children over 3 years of age, but this method is limited by considerable interindividual variability in waveform and latency, as well as a high false-negative rate for visual pathway lesions^10^^.^ In younger children, particularly infants, detecting VGB-induced retinopathy remains difficult due to developmental constraints and the lack of cooperative testing methods. Thus, there is a critical need for sensitive, non-invasive, and objective tools to monitor visual system integrity during VGB therapy in early childhood.

Postmortem studies have reported not only retinal abnormalities but also optic nerve atrophy or even partial absence in patients treated with VGB^11^. These findings are supported by two key anatomical and molecular observations: first, the optic nerve and optic tract originate from retinal ganglion cell axons^12^; and second, both the retina and the visual pathways—including structures like the lateral geniculate body—are rich in GABA_A_, GABA_B_, and GABA_C_ receptors^13–18^^.^ Animal models have shown that VGB increases GABA concentrations by inhibiting GABA degradation, which may contribute to retinal and optic pathway pathology^19^. Therefore, monitoring pathophysiological changes within the visual system offers a promising approach for assessing VGB-induced alterations in children with West syndrome.

On conventional structural MRI sequences, a subset of patients exhibited symmetrical hyperintensity in the thalamus on diffusion-weighted imaging (DWI), while others showed no imaging abnormalities, regardless of the presence or absence of thalamic DWI hyperintensity. The occurrence of symmetrical thalamic hyperintensity on DWI is likely associated with elevated GABA accumulation in the thalamus and visual pathways^20,21^. Despite these findings, little attention has been paid to microstructural changes in the optic nerve, both during VGB treatment and after its discontinuation.

Diffusion tensor imaging (DTI) is a non-invasive imaging modality used to evaluate white matter integrity in vivo, based on the directional diffusivity of water molecules within tissue. Two key DTI parameters—fractional anisotropy (FA) and apparent diffusion coefficient (ADC)—are particularly sensitive in detecting microstructural alterations in nerve fibers^22–24^. Previous studies have applied DTI at the voxel level to identify white matter injuries, including lesions in the optic radiations^25–27^, and to assess optic nerve fiber integrity in patients with optic neuritis^28–30^. However, few investigations have explored the extent of visual pathway alterations or its potential reversibility using DTI in the context of VGB treatment.

This study aimed to evaluate FA and ADC values as sensitive markers for detecting optic nerve injury associated with VGB therapy in children with West syndrome. Receiver operating characteristic (ROC) curve analysis was employed to establish diagnostic thresholds and guide clinical decision-making regarding VGB administration.

## Method

### Clinical data

A total of 35 children diagnosed with West syndrome were retrospectively enrolled from Shenzhen Children’s Hospital between January 2014 and April 2021. Inclusion criteria were: (1) confirmed diagnosis of West syndrome, (2) age less than 6 months at the time of diagnosis, (3) complete clinical records, and (4) high-quality DTI imaging. Exclusion criteria included incomplete clinical data, inability to undergo MRI due to sedation intolerance, and lack of parental consent. The etiology of West syndrome was classified as symptomatic based on clinical and imaging findings. Symptomatic cases included those with tuberous sclerosis.

We hypothesized that symmetrical thalamic hyperintensity on DWI may reflect widespread GABAergic accumulation and serve as a surrogate marker for increased vulnerability of the optic pathway. Thus, patients were grouped based on the presence or absence of thalamic abnormalities. Participants were divided into three groups based on treatment regimen and imaging findings:

Group 1 (n = 11): patients treated with VGB and other ASMs, who showed symmetrical thalamic hyperintensity on DWI. Some of these patients also exhibited additional brain abnormalities (e.g., globus pallidus, substantia nigra) on DWI;

Group 2 (n = 11): patients receiving VGB and other ASMs, but without DWI evidence of symmetrical thalamic hyperintensity;

Group 3 (n = 13): patients treated with ASMs other than VGB, all of whom had normal DWI scans.

In Group 1, all patients discontinued VGB for three months following the detection of symmetrical thalamic hyperintensity. Of these, six patients underwent follow-up DTI to evaluate changes in FA and ADC values.

Clinical data collected for all patients included sex, age at treatment initiation, treatment duration, daily dosage, and cumulative VGB dose. All patients underwent baseline MRI before or at the time of initiating VGB therapy to exclude pre-existing thalamic or optic tract abnormalities. For Group 1, symmetrical thalamic hyperintensity was first identified on MRI obtained within 1–2 weeks after VGB initiation.In addition, perinatal histories—including gestational age, birth weight, Apgar scores, and any complications during delivery—were reviewed for all patients. No significant perinatal complications were recorded among the participants. The study protocol was approved by the Ethics Committee of Shenzhen Children’s Hospital (Approval No. 2021002), and all procedures were conducted in accordance with relevant guidelines and regulations.

### Image acquisitions

All patients underwent conventional MRI scan and DTI. Foam cushions were utilized to reduce head movements. Imaging data were gathered using an 8-channel head coil on a 3 T Siemens scanner (Skyra, Siemens, Germany) at the Shenzhen Children’s Hospital (Shenzhen, China). The localization line was the parallel line at the lower border of the corpus callosum. The parameters for conventional axial T1WI and T2WI:T1WI: section thickness of 5 mm, spacing of 1 mm, TR of 2307 ms, TE of 10.6 ms; T2WI: Fast Spin Echo (FSE) sequence: section thickness of 5 mm, spacing of 1 mm, TR of 4000 ms, TE of 103 ms; sagittal T1WI: section thickness of 5 mm, spacing of 1.5 mm, TR of 200 ms, TE of 2.49 ms. The DTI protocol was using a spin echo planar image sequence with the following parameters: TR/TE = 6800/93 ms, FOV = 220 × 220 mm2, 40 axial slices, slice thickness = 2.5 mm, and in plane resolution = 1.719 × 1.719 mm2. Diffusion weighing was isotropically distributed along 30 directions (b = 1000 s/mm2).

Flash visual evoked potentials (FVEP) were recorded using the MEB-9404C system (Nihon Kohden, Japan). Patients wore LED flash stimulator goggles (Unique Medical, Tokyo, Japan) equipped with 20 red lights and a transparent protective film, allowing real-time monitoring of visual function. Stimulation parameters included a red light source in flashing mode at 1.0 Hz, with a filter bandwidth of 1–100 Hz and fewer than 50 signal averages per recording.

FVEP and electroretinography (ERG) recordings were obtained 2–3 times at each time point, and typical waveforms with minimal interference were selected for analysis. In this study, In line with pediatric FVEP standards, we evaluated N2 (~ 200 ms), P2 (~ 200 ms), and N3 (~ 300 ms) latencies, rather than adult RP-VEP components (N75, P100). Fundus imaging was available for a subset of patients, and one female patient showed peripheral retinopathy (see Fig. 2C). However, this finding was isolated and requires further investigation.

### Imaging processing

Image post-processing was conducted in a blinded manner by an experienced radiologist using the Siemens Syngo workstation. Each measurement was performed three times, and the mean values of the evaluated parameters were used for analysis.

**Fig.1:**
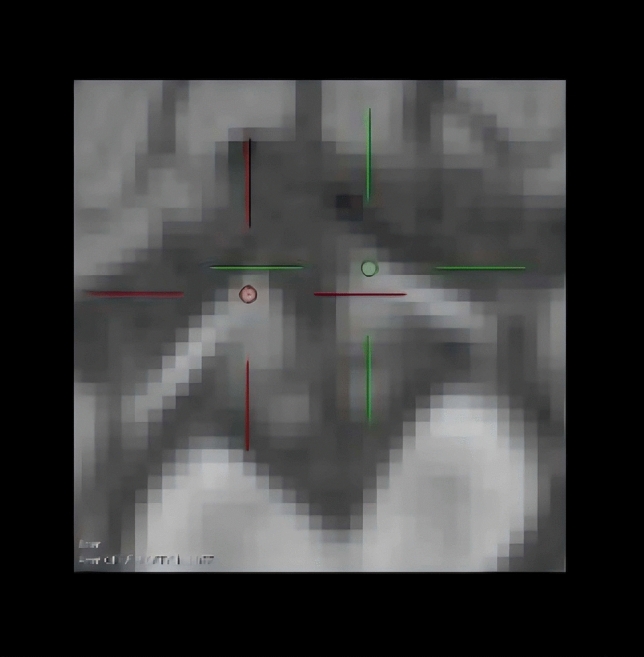
The red color was marked as the picking point of the right optic tract, and the green color was marked as the picking point of the left optic tract.

Region of Interest (ROI) Selection: ROIs were manually placed to capture the center of the optic tract in each slice, with care taken to avoid partial volume effects from adjacent structures. The selected ROI was limited to approximately three pixels in diameter on the cross-sectional plane, ensuring high reproducibility and accuracy. The starting point of each patient’s optic tract was used as a consistent anatomical landmark, and ROIs were placed at a fixed lateral position across all cases. The smallest available voxel size within the workstation was used for delineation (see Fig. 1). The FA and ADC values of bilateral optic nerves were manually measured from axial slices containing the center of the optic nerve. ROIs (~ 3 pixels in diameter) were placed symmetrically and carefully adjusted to avoid partial volume effects. Each ROI was drawn three times and averaged. Image processing was performed using Siemens Syngo.via software. No significant artifacts or limitations (e.g., motion, distortion) affected measurement accuracy.

### Data analyses

Statistical analyses were performed using SPSS software version 25.0 (IBM Corporation, Armonk, NY, USA). The Shapiro–Wilk test was used to assess the normality of data distribution, and the Levene test was employed to evaluate the homogeneity of variances. Variables with non-normal distributions were reported as medians with interquartile ranges (IQRs).

Group comparisons were conducted using one-way analysis of variance (ANOVA), followed by Bonferroni post hoc correction for multiple comparisons where applicable. All statistical tests were two-tailed, and a P-value < 0.05 was considered statistically significant.

Receiver operating characteristic (ROC) curve analysis was used to determine the optimal cut-off value of FA for identifying optic nerve alterations. Statistical graphs were generated using GraphPad Prism version 9.5. All data supporting the findings of this study are provided within the manuscript or the supplementary materials.In addition to group comparisons, we conducted correlation analyses between FA/ADC values and clinical variables (age at MRI, VGB dose, treatment duration). No significant correlations were observed, possibly due to the limited sample size.

## Results

### Clinical manifestations

None of the patients developed new seizure symptoms during VGB treatment.None of the guardians reported overt visual complaints or behavioral signs of visual impairment during the study period. The primary reason for VGB discontinuation in Group 1 was the appearance of symmetrical thalamic hyperintensity on DWI, rather than clinical visual symptoms.

In Group 1, 11 children began VGB therapy between the ages of 9 days and 15 months. The duration of treatment ranged from 9.5 to 17.0 months. The maximum daily dose varied from 67.0 to 108.0 mg/kg/day, and the cumulative VGB dose ranged from 2.2 g to 615.0 g.

In Group 2, 11 children initiated VGB treatment between 3 and 19 months of age. Treatment duration ranged from 7.0 to 19.0 months, with maximum daily doses ranging from 35.7 to 194.0 mg/kg/day. Cumulative VGB doses ranged from 30.0 g to 300.0 g.

In Group 3, 13 children received other anti-seizure medications (ASMs) without VGB to manage seizures. The treatment duration in this group ranged from 9.5 to 18.0 months.

Detailed clinical characteristics of each group are presented in Table 1.

**Table 1:** Clinical Manifestations of Participants.

Group	The first group (VGB&Abnormal imaging)	The second group (VGB&Normal imaging)	The third group (Control)	P-value
Number of the patients	11	11	13	
Sex (Male/Female)	7/4	5/6	7/6	0.865
Start age of medecation (Months)(Median (IQR))	6.0 (1.0–15.0)	5.0 (3.0–19.0)	6.0 (2.0–20.0)	0.998
Duration of medication (Months)(Median (IQR))	11.0 (10.5- 16.0)	11.0 (7.0- 18.5)	11.0 (10.0- 17.0)	0.999
Maximum od daily Dose (mg/kg/d) (Median (IQR))	93 (67.0–100.0)	83 (68.0- 138.0)	None	1.000
Drug Cumulation(g) (Median (IQR))	127.5 (44.6- 158.5)	52.5 (30.0–93.8)	None	0.335

### Conventional imaging features

**Fig.2:**
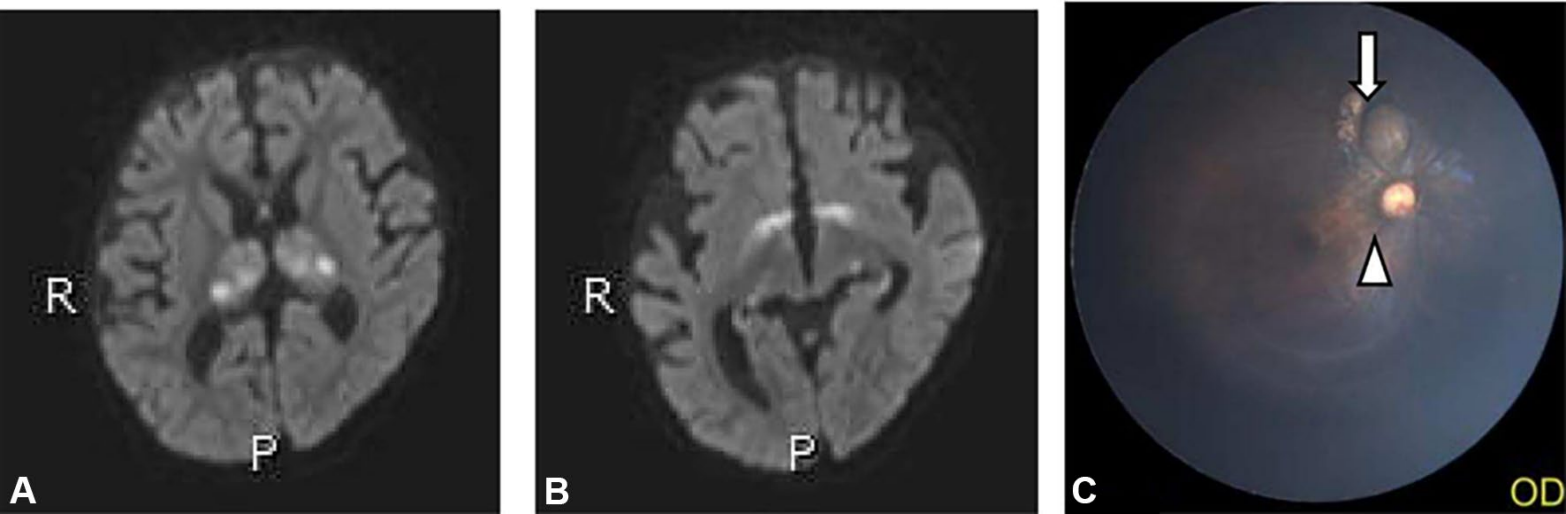
The female patient with West syndrome, was started on VGB at a dose of 75.0 mg/kg per day at the age of six months, with a cumulative VGB dose of approximately 141.5 g. **A**. DWI sequence showed bilateral thalamic symmetrical hyper-intensity, heterogeneous. **B**. DWI sequences showed abnormal hyper-intensity in the bilateral symmetric globus pallidum, medial geniculate body, and lateral geniculate body with slightly less uniform signal. **C**. Long arrows refer to retinopathy, and triangles refer to the optic disc.

In Group 1, diffusion-weighted imaging (DWI) revealed symmetrical hyperintensity in the thalamus, anterior commissure, globus pallidus, substantia nigra, red nucleus, dentate nucleus, and the dorsal brainstem (Fig. 2A and B). Hyperintensity in the medial and lateral geniculate bodies was observed in 8 out of 11 patients (72.7%) on DWI, while conventional MRI sequences showed no corresponding abnormalities.

On conventional sequences, the thalamus exhibited mildly decreased signal intensity on T1-weighted imaging (T1WI) and mildly increased signal intensity on T2-weighted imaging (T2WI) in 7 of 11 patients (63.6%). Similar changes were seen in the globus pallidus in 5 of 11 patients (45.5%). The anterior commissure, dorsal brainstem, dentate nucleus, substantia nigra, and red nucleus appeared normal on conventional imaging.

No intracranial abnormalities were detected in Groups 2 and 3 on either conventional MRI or DWI. Additionally, the optic nerves of all patients appeared normal across all imaging sequences.

### Results of statistical analysis of FA values and ADC values of both lateral optic

The FA and ADC values of the bilateral optic nerves are presented in Table 2. FA values in Group 1 were significantly lower than those in Group 3 (P < 0.05), suggesting microstructural alterations to the optic nerves. Although ADC values in Group 1 were higher than those in the other groups, the differences did not reach statistical significance (P > 0.05).

**Table 2:** FA and ADC values of the bilateral optic nerve for Participants.

Group	The first group (VGB&Abnormal imaging)	The second group (VGB&Normal imaging)	The third group (Control)	df	F	P-value
ROADC (× 10^−6^mm^2^/s) (Median (IQR))	1209.0 (1173.5–1692.5)	1125.0 (990.5–1586.5)	1280.0 (1049.0–1579.0)	2	0.480	0.623
LOADC (× 10^−6^mm^2^/s) (Median (IQR))	1427.0 (1271.5–1624.0)	1435.0 (1309.0–1750.0)	1366.0 (1051.0–1734.0)	2	0.667	0.520
ROFA (Median (IQR))	0.299* (0.270–0.363)	0.463 (0.363–0.518)	0.427 (0.374–0.533)	2	6.173	0.005
LOFA (Median (IQR))	0.297* (0.239–0.326)	0.390 (0.374–0.410)	0.410* (0.371–0.523)	2	9.460	0.001

Notes: *Significant difference between the first and third groups:p < 0.05.

**Fig.3:**
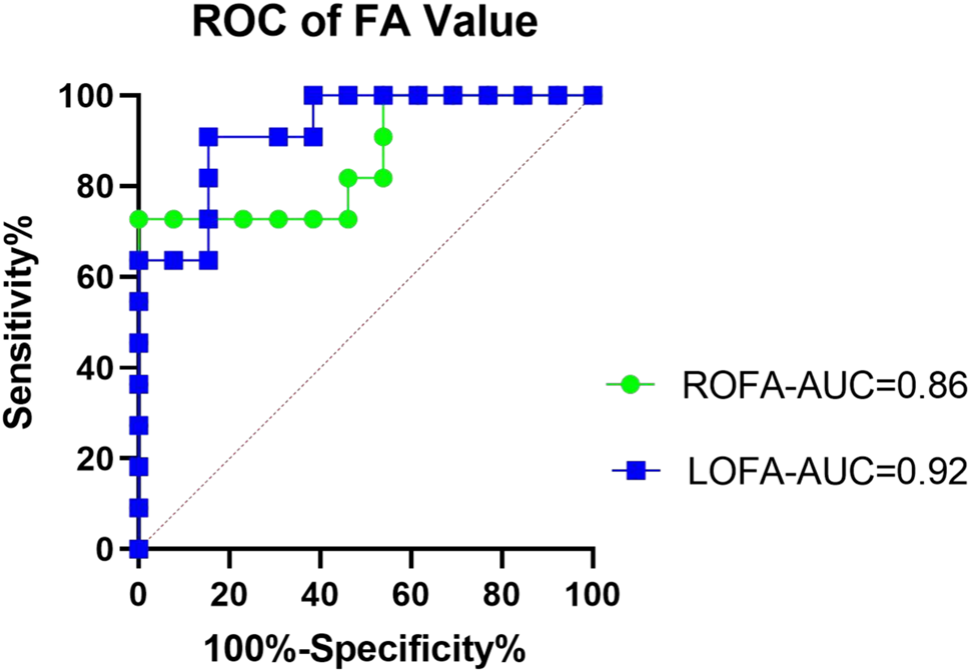
The ROC curve is drawn according to the results, and within the confidence interval, AUC-LOFA is 0.92 and AUC-ROFA is 0.86, the cut-off value 304 is measured.

Following the discontinuation of VGB, FA values in Group 1 showed a significant increase compared to baseline (P < 0.05), indicating partial recovery of optic nerve integrity. To assess the diagnostic utility of FA values for detecting optic nerve alterations, receiver operating characteristic (ROC) curves were constructed. The area under the curve (AUC) was 0.92 for the left optic nerve FA (LOFA) and 0.86 for the right optic nerve FA (ROFA), demonstrating excellent discriminatory performance. An FA cut-off value of 304 yielded a sensitivity of 63.6% and a specificity of 100% for identifying optic nerve impairment (Fig. 3).

IIn the subset of patients from Group 1 who underwent repeat DTI three months after VGB withdrawal, bilateral FA values showed a modest but significant increase compared to values during treatment (Table 3), further supporting the reversibility of VGB-induced changes in the optic nerves.

**Table 3:** Comparison of FA and ADC Values in the Optic Tract of Six Patients while Taking VGB and After Discontinuing VGB for 3 Months.

	VGB	Stop-VGB	Statistic	P value
FA value				
LOFA	0.273. ± 0.064	0.367 ± 0.062	2.68297	0.044
ROFA	0.286 ± 0.020	0.437 ± 0.128	2.56884	0.050
ADC value(× 10^−6^mm^2^/s)				
LOADC	1509.5 ± 310.5	1368.5 ± 254.3	−0.88412	0.417
ROADC	1271.0 ± 311.0	1203.3 ± 241.3	−0.74588	0.489

Notes: VGB for children taking VGB; Stop VGB for discontinued VGB children; LOFA for left optic tract FA value; ROFA for right optic tract FA value; LOADC is the right optic tract ADC value; ROADC is the left optic tract ADC value.

### Flash visual evoked potentials

Flash visual evoked potentials (FVEP) were performed in all participants and demonstrated normal P100 latencies and amplitudes, suggesting intact visual pathway conduction. All children underwent baseline ophthalmologic examinations, including fundus photography and FVEP, which showed no pre-existing visual abnormalities prior to VGB treatment.

## Discussion

VGB is a first-line treatment for patients with West syndrome and has demonstrated efficacy in achieving seizure control. This study offers novel insights into the impact of VGB on optic nerve integrity and its potential reversibility. Specifically, FA values of the optic nerves significantly decreased during VGB treatment, accompanied by symmetrical thalamic hyperintensity on diffusion-weighted imaging (DWI), indicating substantial microstructural alterations.

A key finding of this study is the normalization of FA values within three months following VGB discontinuation, suggesting that VGB-induced microstructural changes in the optic nerves are, at least partially, reversible. Notably, in cases without symmetrical thalamic hyperintensity on DWI, FA values did not significantly differ from those of West syndrome patients who had not received VGB, implying milder or absent optic nerve involvement in these cases. These results suggest that the presence of symmetrical thalamic hyperintensity on DWI may serve as an imaging biomarker for identifying patients at higher risk of VGB-related optic nerve alterations.

Overall, these findings underscore the importance of close monitoring of optic nerve integrity during VGB therapy using advanced imaging techniques such as DWI and FA analysis. Notably, abnormalities were evident on DWI but not on T2WI or FLAIR, possibly reflecting early cytotoxic edema without macroscopic structural alterations—findings consistent with prior reports.The observed reversibility of optic nerve alterations supports the continued use of VGB in managing epilepsy in patients with West syndrome, provided that regular imaging surveillance is performed. This study is distinguished from previous research by demonstrating both the potential reversibility of VGB-induced microstructural changes and the utility of imaging biomarkers in guiding personalized treatment strategies.

### General clinical features and indicators

VGB is a GABA analog with a hydrophilic structure. It is not metabolized by the liver, does not bind to plasma proteins, and is excreted primarily through the kidneys, making it relatively safe for clinical use^27,28^. In this study, the cumulative dose of VGB did not differ between Group 1 and Group 2. The daily dosage of VGB was below the maximum recommended threshold reported in previous studies, suggesting that the occurrence of symmetrical thalamic hyperintensity on DWI was not associated with VGB overdose or cumulative exposure. This finding contrasts with the results of Hussain et al., who observed such changes at doses exceeding 175 mg/kg/day^29^^.^ Our results suggest that the symmetrical DWI abnormalities are more likely related to the maximum daily dose rather than the total cumulative dose.

### Significance of imaging features

Symmetrical hyperintensity in the subthalamic region and basal ganglia was observed on DWI following VGB administration and resolved after drug withdrawal. This finding suggests that the abnormal hyperintensity may be due to cytotoxic edema resulting from VGB metabolism. This is consistent with previous reports demonstrating cytotoxic edema in pathological sections of rat brains after VGB treatment[^30], as well as in human brain studies^31–33^. The differential distribution of GABA_A_, GABA_B__, and GABA_C_ receptors throughout the central nervous system (CNS) may contribute to these findings. In particular, impaired GABA degradation has been implicated in the thalamus and other related anatomical structures, although the specific mechanisms remain unclear^3–5^.

In this study, we observed for the first time abnormal signals in gray matter nuclei including the substantia nigra, red nucleus, medial geniculate body, and lateral geniculate body. These lesions appear to be analogous to those in the thalamus and are also suggestive of cytotoxic edema.

DTI has proven useful in evaluating fiber tract integrity in various brain regions, including the optic nerve, optic tract, and optic radiation. FA, a key parameter in DTI, reflects myelin integrity, axonal density, and fiber orientation^28^. ADC, another important DTI metric, measures the Brownian motion of water molecules in vivo and reflects pathophysiological changes in the microstructure of neural tissue^12,34^. Therefore, DTI provides a valuable tool for assessing the integrity of visual fiber tracts.

We aimed to utilize DTI for routine monitoring of optic nerve injury, particularly because some patients appear to be highly sensitive to VGB, with optic nerve alterations occurring even at relatively low doses. In such cases, although no overt clinical symptoms were present, DTI revealed subclinical nerve injury. Thus, DTI can serve as an early warning tool to help determine the appropriate VGB dosage.

In this study, FA values of the optic nerve fiber tracts in Group 1 were significantly lower than those in the other groups, suggesting severe optic nerve alterations associated with symmetrical thalamic hyperintensity on DWI after VGB administration. This may be related to VGB’s suppression of cortical excitability and seizure activity, as well as its role in reducing GABA degradation in the retina, visual pathways, and thalamus^19^.

Importantly, FA values returned to normal within three months of VGB withdrawal and were no longer significantly different from baseline, suggesting that the cytotoxic changes in nerve fibers are reversible and may be independent of irreversible visual impairment^15^. Therefore, we propose that upon identification of symmetrical thalamic hyperintensity on DWI, temporary discontinuation of VGB should be considered. Restoration of microstructural integrity can be expected within approximately three months, after which VGB treatment may be resumed.

Some patients in this study discontinued VGB upon detection of DWI abnormalities and later resumed treatment once the imaging abnormalities resolved. While the anti-seizure efficacy of VGB remained robust, its precise effects on the optic nerve remain unclear. Notably, some patients without symmetrical thalamic hyperintensity on DWI still exhibited mildly reduced FA values, suggesting that VGB may be continued in seizure-free patients without overt imaging abnormalities.

This study also identified an FA cut-off value of 304, below which optic nerve alterations may be suspected. Although DTI has been widely applied to assess nerve fiber injury in conditions such as multiple sclerosis and neuromyelitis optica, its use in evaluating irreversible visual field deficits associated with VGB remains limited^35^.

In this study, ADC values of the bilateral optic nerves were elevated, suggesting altered water molecule diffusion, potentially due to microstructural changes. However, the differences were only partially significant and not consistent between the left and right optic nerves. This inconsistency implies that ADC may not be sensitive enough to detect early microstructural changes in the optic tract, possibly limiting its utility for early diagnosis^35^.

The variability in optic tract involvement and its reversibility following VGB discontinuation may be attributable to individual differences in VGB-associated metabolism (VGBAM). Some patients appear to be particularly sensitive to VGB, showing signs of optic nerve alterations even at lower doses. VGB maintains elevated GABA levels in the CNS, and abnormalities may persist until the drug is fully cleared and GABA metabolism is restored. The duration of abnormal signal presence may therefore vary depending on individual differences in GABA metabolism.While elevated GABA has been proposed as a contributing factor to white matter changes, studies suggest that mitochondrial dysfunction may play a more central role. VGB inhibits GABA transaminase within mitochondria, potentially impairing nucleotide metabolism and myelin integrity via disrupted dNDP-to-dNTP conversion^36^. Electron microscopy findings further support the presence of white matter spongiosis and myelin vacuolation in both animal models and postmortem human studies^37,38^.

Visual evoked potentials (VEPs) were essentially normal across all patient groups in this study. However, VEP lacks the ability to localize optic nerve alterations and has known limitations in detecting subclinical changes^39^. In contrast, FA measurements via DTI offer greater sensitivity in detecting microstructural alterations to optic nerve fibers, making DTI a superior tool for monitoring VGB-related optic neuropathy. In the subset of children who resumed VGB after resolution of imaging abnormalities, treatment was reinstated due to seizure recurrence and poor response to alternative ASMs, highlighting the balance between seizure control and visual safety.

### Deficiency

This is a single center study, and VGBAM is rare to find out as Hussain et.al reported^29^, so that we cannot have a large number of patients to study. DTI imaging follow-up was performed in only 6 patients owing to patient partial compliance.

### Summary

This study demonstrates that VGB-induced optic nerve alterations in children with West syndrome can be effectively monitored using FA values derived from DTI. The most severe alterations was observed in patients presenting with symmetrical thalamic hyperintensity on DWI, although no corresponding clinical symptoms were evident, and the underlying mechanism remains unclear. Notably, FA values returned to baseline levels following the discontinuation of VGB, suggesting that treatment may be cautiously resumed under close monitoring.

These findings provide important evidence for the early detection and management of VGB-related optic nerve toxicity and offer a practical framework for ensuring the safe use of VGB in children with West syndrome.

## Supplementary Information

Supplementary Information 1.

Supplementary Information 2.

## Data Availability

Data is provided within the manuscript or supplementary information files.

## Abbreviations

VGB: Vigabatrin
FA: Fractional anisotropy
ADC: Apparent diffusion coefficient
DTI: Diffusion tensor imaging
DWI: Diffusion-weighted imaging
FVEP: Flash visual evoked potential
ERG: Electroretinography
ASM: Anti-seizure medication

## References

[1] Ben-Menachem E.Vigabatrin. *Epilepsia*. .36(Suppl 2): S95–S104.6.10.1111/j.1528-1157.1995.tb06003.x (1995). 10.1111/j.1528-1157.1995.tb06003.x 8784218

[2] Cubells, J. F. et al. In vivo action of enzyme-activated irreversible inhibitors of glutamic acid decarboxylase and gammaaminobutyric acid transaminase in retina vs.brain. *J. Pharmacol. Exp. Ther.* **238**, 508–514 (1986). 3735130

[3] Neal, M. J. et al. Immunocytochemical evidence that vigabatrin in rats causes GABA accumulation in glial cells of the retina. *Neurosci Lett.* **98**, 29–32. 10.1016/0304-3940(89)90368-6 (1989). 2710396 10.1016/0304-3940(89)90368-6

[4] Sills, G. J. et al. Vigabatrin, but not gabapentin or topiramate, produces concentration-related effects onenzymes and intermediates of the GABA shunt in rat brain and retina. *Epilepsia* **44**, 886–892. 10.1046/j.1528-1157.2003.04203.x (2003). 12823570 10.1046/j.1528-1157.2003.04203.x

[5] Fejerman, N. et al. Vigabatrin as a first-choice drug in the treatment of West syndrome. *J. Child Neurol.*10.1177/088307380001500304 (2000). 10757471 10.1177/088307380001500304

[6] Riikonen, R. Combination therapy for treatment of infantile spasms. *The Lancet Neurol.* **16**(1), 19–20. 10.1016/s1474-4422(16)30276-9 (2020). 10.1016/S1474-4422(16)30276-9 27838191

[7] Hébert-Lalonde, N. et al. Electrophysiological Evidences of Visual Field Alterations in Children Exposed to Vigabatrin Early in Life. *Pediatr. Neurol.* **59**, 47–53. 10.1016/j.pediatrneurol.2016.03.001 (2016). 27105764 10.1016/j.pediatrneurol.2016.03.001

[8] Wild, J. M., Aljarudi, S., Smith, P. E. & Knupp, C. The topographical relationship between visual field loss and peripapillary retinal nerve fibre layer thinning arising from long-term exposure to vigabatrin. *CNS Drugs* **33**, 161–173 (2019). 30637668 10.1007/s40263-018-0583-8

[9] Kjellström, U., Andréasson, S. & Ponjavic, V. Attenuation of the retinal nerve fibre layer and reduced retinal function assessed by optical coherence tomography and full-field electroretinography in patients exposed to vigabatrin medication. *Acta Ophthalmol.* **92**, 149–157. 10.1111/aos.12030 (2014). 23387307 10.1111/aos.12030

[10] Hawker, M. J. & Astbury, N. J. The ocular side effects of vigabatrin (Sabril): Information and guidance for screening. *Eye* **22**(9), 1097–1098. 10.1038/eye.2008.139 (2018). 10.1038/eye.2008.139 18497834

[11] Ravindran, J. et al. Visual field loss associated with vigabatrin:pathological correlations. *J. Neurol. Neurosurg. Psychiatry.* **70**, 787–789. 10.1136/jnnp.70.6.787 (2001). 11385015 10.1136/jnnp.70.6.787 PMC1737373

[12] Silverthorn, D. U. *Human Physiology* 4th edn. (Pearson Education Inc., 2009).

[13] Bowery, N. G., Hudson, A. L. & Price, G. W. GABAA, and GABAB receptor site distribution in the rat central nervous system. *Neuroscience* **20**, 365–383. 10.1016/0306-4522(87)90098-4 (1987). 3035421 10.1016/0306-4522(87)90098-4

[14] Lukasiewicz, P. D., Maple, B. R. & Werblin, F. S. A novel GABA receptor on bipolar cell terminals in the tiger salamander retina. *Neurosci.* **14**, 1201–1212 (1994). 10.1523/JNEUROSCI.14-03-01202.1994 PMC6577582 8120620

[15] Vaquero, C. F. & de la Villa, P. Localisation of the GABA_C_ receptors at the axon terminal of the rod bipolar cells of the mouse retina. *Neurosci. Res.* **35**, 1–7 (1999). 10555158 10.1016/s0168-0102(99)00050-4

[16] Euler, T. & Masland, R. H. Light-evoked responses of bipolar cells in a mammalian retina. *Neurophysiol.* **83**, 1817–1829 (2000). 10.1152/jn.2000.83.4.1817 10758094

[17] Pattnaik, B., Jellali, A., Sahel, J., Dreyfus, H. & Picaud, S. GABA_C_ receptors are localized with microtubulc-associated protein 1B in mammalian conephotoreceptors. *J. Neurosci.* **20**, 6789–6796 (2000). 10995822 10.1523/JNEUROSCI.20-18-06789.2000 PMC6772813

[18] Walters, D. C. et al. Metabolomic analysis of vigabatrin (VGB)-treated mice: GABA-transaminase inhibition significantly alters amino acid profiles in murine neural and non-neural tissues. *Neurochem. Int.* **05**, 125. 10.1016/j.neuint.2019.02.015 (2019). 10.1016/j.neuint.2019.02.015 PMC6414070 30822440

[19] Guitaries, R. P. et al. A multimodal evaluation of microstructural white matter damage in spinocerebellar ataxia type 3. *Mov. Disord.* **28**(8), 1125–1132. 10.1002/mds.25451 (2013). 23553599 10.1002/mds.25451

[20] Miller, S. P. et al. Abnormal Brain Development in Newborns with Congenital Heart Disease. *New England J. Medic.* **357**(19), 1928–1938. 10.1056/nejmoa067393 (2007). 10.1056/NEJMoa067393 17989385

[21] Croall, I. D. et al. Using DTI to assess white matter microstructure in cerebral small vessel disease (SVD) in multicentre studies. *Clin. Sci.* **131**(12), 1361–1373. 10.1042/cs20170146 (2017). 10.1042/CS20170146 PMC5461938 28487471

[22] Alves, C. et al. The retinal ganglion cell layer predicts normal-appearing white matter tract integrity in multiple sclerosis: A combined diffusion tensor imaging and optical coherence tomography approach. *Hum. Brain Mapp.* **39**, 1712–1720. 10.1002/hbm.23946 (2018). 29334156 10.1002/hbm.23946 PMC6866258

[23] Balk, L. J. et al. Bidirectional transsynaptic axonal degeneration in the visual pathway in multiple sclerosis. *J. Neurol. Neurosurg. Psychiatry.* **86**, 419–424. 10.1136/jnnp-2014-308189 (2015). 24973342 10.1136/jnnp-2014-308189

[24] Klistorner, A. et al. Axonal loss of retinal neurons in multiple sclerosis associated with optic radiation lesions. *Neurology* **82**, 2165–2172. 10.1212/WNL.0000000000000522 (2014). 24838786 10.1212/WNL.0000000000000522 PMC4113462

[25] Xc H.Reconstruction and dissection of the entire human visual pathway using diffusion tensor MRI. *Frontiers in Neuroanatomy.*10.3389/fnana.2010.00015 (2010). 10.3389/fnana.2010.00015 PMC2859811 20428499

[26] Wang, L., Das, S. & Yang, H. DTI of great occipital nerve neuropathy: an initial study in patients with cervicogenic headache. *Clin. Radiol.* **74**(899), e1–e899 (2019). 10.1016/j.crad.2019.07.025 31495544

[27] Juhász, C. et al. Prolonged vigabatrin treatment modifies developmental changes of GABA(A)-receptor binding in young children with epilepsy. *Epilepsia*10.1046/j.1528-1157.2001.05401.x (2001). 11737167 10.1046/j.1528-1157.2001.05401.x

[28] Elwes, R. D. & Binnie, C. D. Clinical pharmacokinetics of newer antiepileptic drugs Lamotrigine vigabatrin gabapentin and oxcarbazepine. *Clin. Pharmacokinet.* **30**(6), 403–415. 10.2165/00003088-199630060-00001 (1996). 8792055 10.2165/00003088-199630060-00001

[29] Patsalos, P. N. & Perucca, E. Clinically important drug interactions in epilepsy: general features and interactions between antiepileptic drugs. *Lancet Neurol.* **2**(6), 347–356. 10.1016/s1474-4422(03)00409-5 (2003). 12849151 10.1016/s1474-4422(03)00409-5

[30] Hussain, S. A. et al. Risk of vigabatrin-associated brain abnormalities on MRI in the treatment of infantile spasms is dose-dependent. *Epilepsia*10.1111/epi.1371219 (2017). 28230253 10.1111/epi.13712

[31] Pearl, P. L. et al. White matter spongiosis with vigabatrin therapy for infantile spasms. *Epilepsia* **59**(4), e40–e44. 10.1111/epi.14032 (2018). 29473152 10.1111/epi.14032

[32] Schuitema, I. et al. Accelerated Aging, Decreased White Matter Integrity, and Associated Neuropsychological Dysfunction 25 Years After Pediatric Lymphoid Malignancies. *J. Clin. Oncol.* **31**(27), 3378–3388. 10.1200/jco.2012.46.7050 (2013). 23960182 10.1200/JCO.2012.46.7050

[33] Aliotta E, Nourzadeh H, Batchala P P. et al. Molecular Subtype Classification in Lower-Grade Glioma with Accelerated DTI. *American Journal of Neuroradiology*.2019.10.3174/ajnr.a6162 10.3174/ajnr.A6162 PMC7048441 31413006

[34] Hana, A. et al. DTI of the Visual Pathway - White Matter nerve and Cerebral Lesions. *J. Visualized Exp.*10.3791/51946 (2014). 10.3791/51946 PMC4828020 25226557

[35] Wan, H. et al. Diffusion-weighted imaging helps differentiate multiple sclerosis and neuromyelitis optical-related acute optic neuritis. *J. Magn Reson Imag.*10.1002/jmri.25528 (2017). 10.1002/jmri.25528 27859858

[36] Arnaud B ,Ping Wu, Francesco B, et al. The GABA transaminase, ABAT, is essential for mitochondrial nucleoside metabolism. .Cell Metab, 2015, 21: 10.1016/j.cmet.2015.02.008 10.1016/j.cmet.2015.02.008 PMC4757431 25738457

[37] Phillip LP,Annapurna P,Sanjay PP, et al. White matter spongiosis with vigabatrin therapy for infantile spasms. .Epilepsia, 2018, 59: 10.1111/epi.14032 10.1111/epi.14032 29473152

[38] Cohen JA, Fisher RS, Brigell MG, et al. The potential for vigabatrin-induced intramyelinic edema in humans. .Epilepsia, 2000, 41:10.1111/j.1528-1157.2000.tb00134.x 10.1111/j.1528-1157.2000.tb00134.x 10691111

[39] Cammann R. Use of visual evoked potentials in neurology--a review. II. Zentralblatt fur. *Neurochirurgie.*1985. 4013563

