# Supplementary material for: Microstructural injury to the optic nerve with vigabatrin treatment in West syndrome: A DTI study
Source: Sci Rep. 2025 Jul 16;15:25754. doi: 10.1038/s41598-025-06336-8 (PMC12267420; doi:10.1038/s41598-025-06336-8)
Supplement: Supplementary file 2 — Supplementary Information 2. [file 41598_2025_6336_MOESM2_ESM.docx]

### Author Agreement

All authors of this manuscript have made significant contributions to the work and are in agreement with the content of the manuscript. Each author has participated sufficiently in the research to take public responsibility for appropriate portions of the content. Specifically, the contributions of the authors are as follows:

1. **[Hu-Junjie]**: Conception and design of the study, data analysis, and drafting the manuscript.
2. **[Chen-Li,Zhang-Gongwei,Li-Yilian]**: Acquisition of data, interpretation of findings, and critical revision of the manuscript.
3. **[Fang-Yu,Zhang-Huiting]**: Data processing, technical support for DTI imaging, and final approval of the manuscript.
4. **[Liao-Jianxiang&Zhao-Cailei]**: Have the same contribution of supervision of the research process, critical intellectual input, and approval of the final version.

**
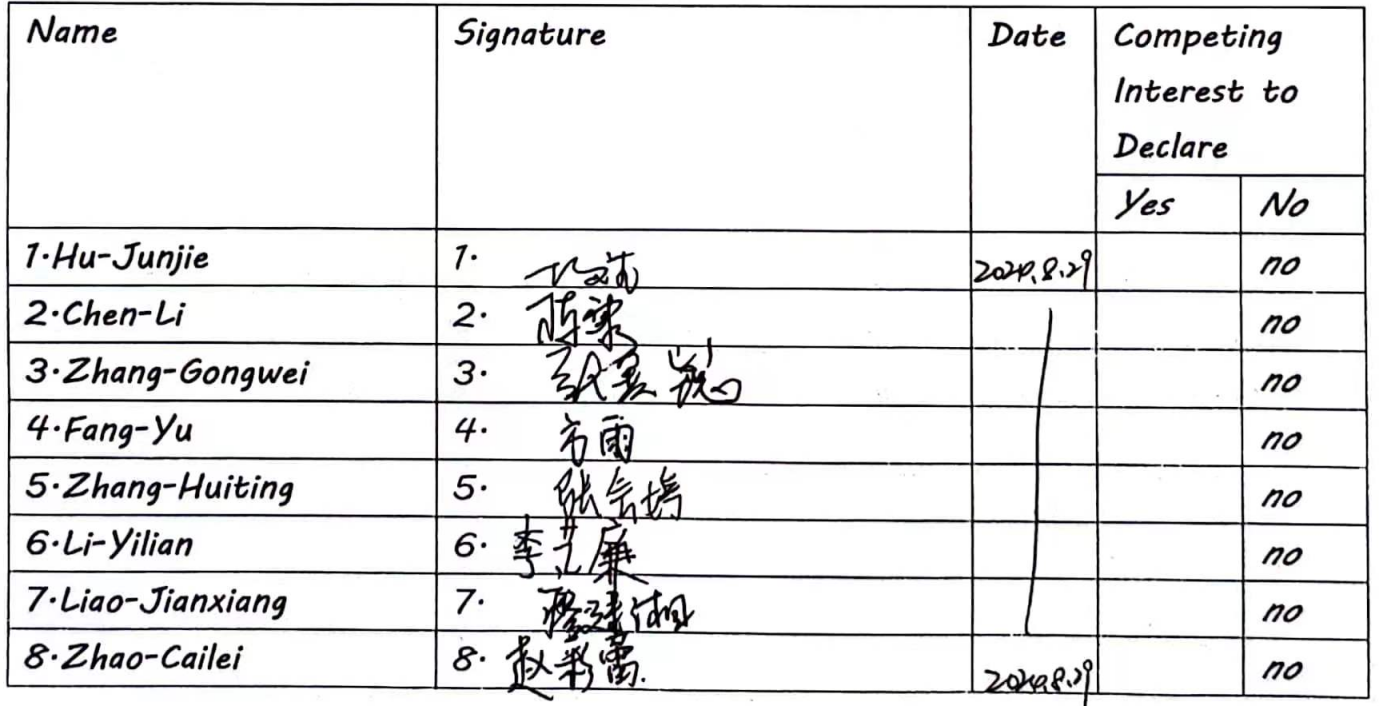
**

The manuscript has not been submitted to or published in any other journal. All authors have read and approved the final version of the manuscript and agree to its submission to [EUROPEAN JOURNAL OF PAEDIATRIC NEUROLOGY].

Furthermore, the authors confirm that all appropriate ethical guidelines have been followed, and necessary approvals were obtained for conducting the research.
